# Metabarcoding reveals low prevalence of microsporidian infections in castor bean tick (*Ixodes ricinus*)

**DOI:** 10.1186/s13071-022-05150-9

**Published:** 2022-01-15

**Authors:** Artur Trzebny, Justyna Liberska, Anna Slodkowicz-Kowalska, Miroslawa Dabert

**Affiliations:** 1 Molecular Biology Techniques Laboratory, Faculty of Biology, Adam Mickiewicz University, Poznan, Poland; 2 Department of Biology and Medical Parasitology, Faculty of Medicine I, University of Medical Sciences, Poznan, Poland

**Keywords:** Microsporidia, Vector-borne diseases, Zoonoses, Parasitic diseases, Microsporidiosis, DNA metabarcoding, Ticks, *Ixodes ricinus*, DNA barcoding, Molecular diagnostics

## Abstract

**Background:**

Microsporidia is a large group of eukaryotic obligate intracellular spore-forming parasites, of which 17 species can cause microsporidiosis in humans. Most human-infecting microsporidians belong to the genera *Enterocytozoon* and *Encephalitozoon*. To date, only five microsporidian species, including *Encephalitozoon*-like, have been found in hard ticks (Ixodidae) using microscopic methods, but no sequence data are available for them. Furthermore, no widespread screening for microsporidian-infected ticks based on DNA analysis has been carried out to date. Thus, in this study, we applied a recently developed DNA metabarcoding method for efficient microsporidian DNA identification to assess the role of ticks as potential vectors of microsporidian species causing diseases in humans.

**Methods:**

In total, 1070 (493 juvenile and 577 adult) unfed host-seeking *Ixodes ricinus* ticks collected at urban parks in the city of Poznan, Poland, and 94 engorged tick females fed on dogs and cats were screened for microsporidian DNA. Microsporidians were detected by PCR amplification and sequencing of the hypervariable V5 region of 18S rRNA gene (18S profiling) using the microsporidian-specific primer set. Tick species were identified morphologically and confirmed by amplification and sequencing of the shortened fragment of cytochrome *c* oxidase subunit I gene (mini-COI).

**Results:**

All collected ticks were unambiguously assigned to *I. ricinus*. Potentially zoonotic *Encephalitozoon intestinalis* was identified in three fed ticks (3.2%) collected from three different dogs. In eight unfed host-seeking ticks (0.8%), including three males (1.1%), two females (0.7%) and three nymphs (0.7%), the new microsporidian sequence representing a species belonging to the genus *Endoreticulatus* was identified.

**Conclusions:**

The lack of zoonotic microsporidians in host-seeking ticks suggests that *I*. *ricinus* is not involved in transmission of human-infecting microsporidians. Moreover, a very low occurrence of the other microsporidian species in both fed and host-seeking ticks implies that mechanisms exist to defend ticks against infection with these parasites.

**Graphical abstract:**

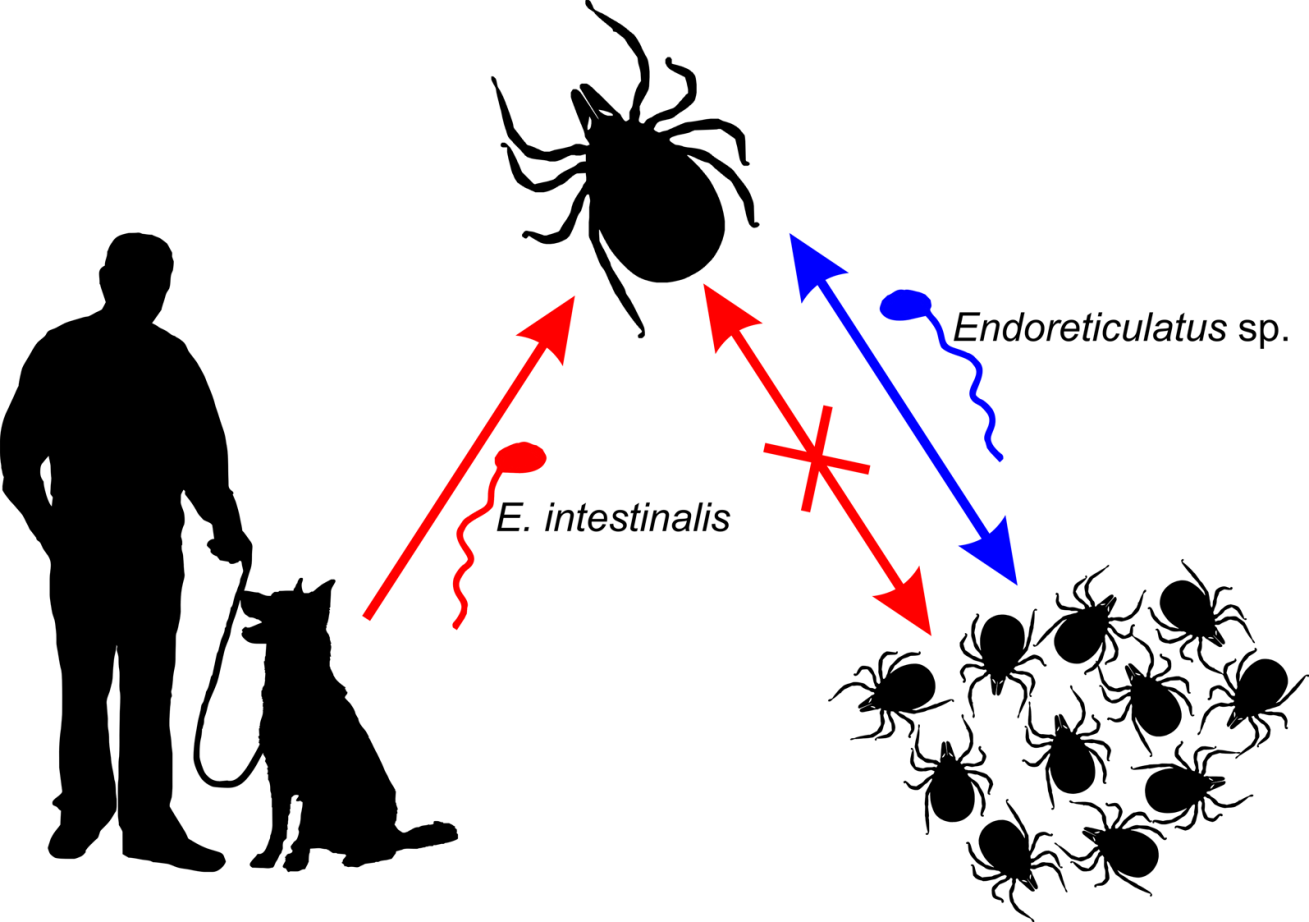

**Supplementary Information:**

The online version contains supplementary material available at 10.1186/s13071-022-05150-9.

## Background

Microsporidia is a group of obligate, intracellular spore-forming eukaryotic parasites. The phylum contains more than 1500 described species belonging to at least 200 genera that infect virtually all animal phyla, including protists [1, 2]. To date, 17 species belonging to eight genera and one holding genus (Microsporidium) were found to cause a wide range of clinical human diseases [3]. In immunocompromised patients, microsporidian infections cause various clinical manifestations, ranging from localized to disseminated, depending on the causative species and host immune status [4]. Until the AIDS pandemic, microsporidiosis was rarely recognized in humans; however, the prevalence of microsporidian infections in case of large infection outbreaks or in HIV-positive patients can reach almost 70% [5]. This shows that, as opportunistic parasites, microsporidians can pose a serious threat, especially when they co-occur with another pathogenic agent. The most common microsporidium infecting humans is *Enterocytozoon bieneusi*, followed by three of four *Encephalitozoon* species, including *E. cuniculi*, *E. hellem* and *E. intestinalis* [6–8]. In addition, spores of *E. bieneusi* and *Encephalitozoon* spp. have been detected in feces of companion animals, including dogs, cats and horses (for review see [6]).

Ticks (Ixodida) are obligate hematophagous arthropods that transmit a wide variety of zoonotic pathogens [9–11]. Among them, the castor bean tick (*Ixodes ricinus*) is the major vector of causative agents of viral, bacterial and protozoan zoonotic diseases in Europe [12]. Moreover, urban green areas may act as pathogen exchange hot spots because of increasing populations of potential reservoirs of tick-borne pathogens and the exposure of humans and domesticated animals to the infected ticks [13, 14].

Knowledge about microsporidian infections in ticks is sparse. To date, only five microsporidian species have been found in these hosts (Table 1). Among them, the *Encephalitozoon*-like species was detected in fed females of *Anocentor nitens* and *Amblyomma cajennense* (Ixodidae) [15, 16]. Screening of microsporidians in other hard ticks, i.e., *Dermacentor marginatus*, *D. reticulatus, I*. *ricinus* and *I. persulcatus*, revealed the presence of three species belonging to the genus *Nosema* [17–24]. Another species of the genus *Nosema* was found in the only examined representative of soft ticks, *Ornithodoros parkei* (Argasidae) [25]. However, all microsporidians found to date in ticks were detected morphologically; none of those species was confirmed by DNA sequencing.

**Table 1:** Summary of published reports on the microsporidian species identified in ticks

Tick species	Tick sex	Feeding behavior	Microsporidian species	Microsporidian positive/tested	References
*Amblyomma cajennense*	Female	Fed on rabbits	*Encephalitozoon*-like	3/10	[15]
*Anocentor nitens*	Female	Fed on rabbits	*Encephalitozoon*-like	5/10	[15]
*Anocentor nitens*	Female	Fed on a horse	*Encephalitozoon*-like	2/7	[16]
*Ornithodoros parkei*	n/a	Fed on mice	*Nosema parkei*	24/130	[25]
*Dermacentor marginatus*	Female	Questing	*Nosema slovaca*	1/370	Following [17]
*Dermacentor reticulatus*	Female	Questing	*Nosema slovaca*	5/1753	[18]
*Dermacentor reticulatus*	Female	Questing	*Nosema slovaca*	1/ n/a	[19]
*Dermacentor reticulatus*	Male	Questing	*Nosema slovaca*	1/1238	[18]
*Dermacentor reticulatus*	Male	Questing	*Nosema slovaca*	1/1363	[20]
*Ixodes persulcatus*	n/a	Questing	*Nosema*-like	3/194	[21]
*Ixodes ricinus*	n/a	Questing	*Nosema*-like	5/13	[22]
*Ixodes ricinus*	Female	Questing	*Nosema slovaca*	4/729	[20]
*Ixodes ricinus*	Female	Questing	*Nosema slovaca*	1/ n/a	[23]
*Ixodes ricinus*	Female	Questing	*Unikaryon (Nosema) ixodis*	1/ n/a	[24]
*Ixodes ricinus*	Female	Questing	*Unikaryon (Nosema) ixodis*	1/ n/a	Following [17]
*Ixodes ricinus*	Female	Questing	*Unikaryon (Nosema) ixodis*	17/162	Following [17]
*Ixodes ricinus*	Male	Questing	*Unikaryon (Nosema) ixodis*	2/217	Following [17]
*Ixodes ricinus*	Male	Questing	*Unikaryon (Nosema) ixodis*	2/ n/a	[24]

*n/a* not available

Over the past years, molecular data have proven their usefulness in many fields of biology, including parasitology. Amplification of marker genes in combination with DNA sequencing is widely used for the rapid screening of multiple samples to detect specific infection agents. In addition, major advances in large-scale DNA sequencing have enabled the development of DNA metabarcoding approaches for biodiversity profiling in which different species are detected simultaneously using group-specific or universal PCR primers [26, 27]. Such approaches have already been applied also to the detection of microsporidian biodiversity [28–30]. In this article, we used the DNA metabarcoding approach to examine the prevalence and diversity of microsporidians in *I. ricinus* to assess the role of ticks as potential vectors of microsporidian species causing diseases in humans.

## Methods

### Tick sampling

**Fig. 1:**
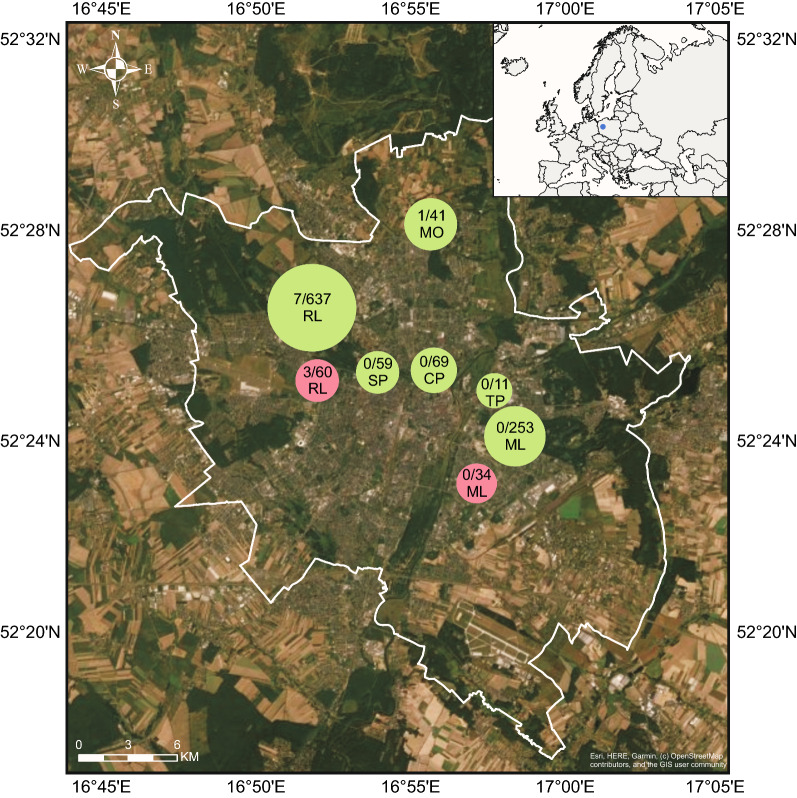
Sampling places of unfed host-seeking ticks (red circles) and feeding (green circles) ticks in the city of Poznan (blue dot on the Europe map). The size of circle corresponds to the number of analyzed ticks. Numeric value inside the circles: number of microsporidian-positive ticks/number of analyzed ticks. For sampling place characteristics, see Additional file 1: Table S1 and Additional file 2: Table S2

In total, 1164 ticks were tested in this study. The sample of host-seeking ticks included 493 juveniles (30 larvae, 463 nymphs) and 577 adults (291 females, 286 males) that were collected from May to June 2017 and April to May 2018 by dragging and flagging at six recreational parks in the city of Poznan, Poland (Fig. 1 and Additional file 1: Table S1). Dragging was conducted by passing the cotton drag (1 m^2^) over the ground level vegetation, while flagging was done by waving over (1 m^2^) and through the higher vegetation (ca. 50–100 cm). Collection sessions were performed between 10:00 am and 7:00 pm. Additionally, 94 engorged females removed from pet animals were provided by veterinary clinics in 2013 and 2016 (Fig. 1; Additional file 2: Table S2).

All ticks were preserved in 96% ethanol until DNA extraction. Tick species was initially identified by morphological traits and confirmed by DNA barcoding using cytochrome *c* oxidase subunit I (COI) amplification and NGS sequencing as described below.

### DNA extraction

Engorged ticks were placed separately in 2-ml tubes containing Lysing Matrix A (MP Biomedicals, USA). After the addition of 360 μl of ATL lysis buffer (Qiagen, Germany), samples were homogenized twice for 30 s at 6.0 ms^−1^ using a FastPrep-24 homogenizer (MP Biomedicals, USA). Proteinase K (Bio Basic, Canada) was added to the samples to the final concentration of 0.2 mg ml^−1^ and then samples were incubated for 48 h at 56 °C with shaking. Subsequently, 100 μl of the lysate was used to extract total genomic DNA using the DNeasy Blood and Tissue Kit (Qiagen) according to the manufacturer’s instructions.

From unfed host-seeking ticks, total genomic DNA was extracted using ammonium hydroxide method [31]. Individual ticks were crushed using a metal stylus in 10 μl of 0.7 M ammonium hydroxide solution (POCH S.A., Poland) and then the next 90 μl of the solution was added. Samples were incubated for 20 min at 99 °C, and then the tubes were opened and left under the same conditions to concentrate the lysate to about 50 μl. Next, the samples were centrifuged for 5 min at 14.100 ×*g*, and the supernatant was collected to fresh tubes. DNA extracts were additionally purified from PCR inhibitors using CentriPure Gel Filtration Plates filled with Zoladex (Biotech GmbH, Germany), according to the manufacturer’s protocol.

DNA isolates from adults were analyzed individually, while those from nymphs and larvae were pooled into groups of three individuals. The samples included in the microsporidian-positive pool were re-analyzed separately in a second round of PCR amplification and NGS sequencing. Negative controls from blank DNA extractions and PCR reagents also were amplified and sequenced.

### Amplification of mini-COI and 18S rDNA for NGS sequencing

DNA barcode covering 322 bp from the 5′ end of the COI gene (mini-COI) was amplified using bcdF01 (CATTTTCHACTAAYCATAARGATATTGG) [32] and bcdR06 (GGDGGRTAHACAGTYCAHCCNGT) [29] primers tailed at 5′ ends with dual-indexed adapters (forward tail: CCATCTCATCCCTGCGTGTCTCCGACTCAG-index-GAT, reverse tail: CCTCTCTATGGGCAGTCGGTGAT-index) for sequencing using the Ion Torrent system (Life Technologies, USA). PCR amplification was performed in a 5-µl volume containing Hot FIREPol DNA Polymerase (Solis BioDyne, Estonia), 0.25 µM of each tailed primer and 1 µl of template DNA. Amplification program was as follows: 12 min at 95 °C, followed by 35 cycles of 15 s at 95 °C, 30 s at 50 °C and 45 s at 72 °C, with a final extension step at 72 °C for 5 min.

The hypervariable V5 region of 18S rRNA gene (18S rDNA) was amplified in two technical replications using the microsporidian-specific primer set CM-V5F (GATTAGANACCNNNGTAGTTC) and CM-V5R (TAANCAGCACAMTCCACTC) [29]. PCR reactions were done in a total volume of 10 µl containing Hot FIREPol DNA Polymerase, 0.25 µM of each tailed primer and 1 µl of template DNA. Amplification program was as follows: 12 min at 95 °C, followed by 40 cycles of 15 s at 95 °C, 30 s at 50 °C and 30 s at 72 °C, with a final extension step at 72 °C for 5 min.

### Library construction and NGS sequencing

The 18S rDNA and mini-COI libraries were prepared separately. For each PCR reaction, 3 µl was electrophoresed on a 2% agarose gel to check amplification efficiency. Then, the amplicons were pooled and purified using 2% E-Gel SizeSelect II Agarose Gels system (Invitrogen, USA), according to the manufacturer’s protocol. DNA concentration and fragment length distribution of the libraries were established using High Sensitivity D1000 Screen Tape assay on 2200 Tape Station system (Agilent Technologies, USA). For the emulsion PCR, the 18S rDNA and mini-COI libraries were pooled in the 10:1 ratio. Emulsion PCR and sequencing were carried out using the Ion Torrent One Touch System II, the Ion 520 and Ion 530 Kit-OT2, Ion 530 chip and the S5 system (Life Technologies) according to the manufacturer's instructions.

### Read processing and data analysis

Raw sequence data were pre-filtered by Ion Torrent Suite software version 5.10.1 (Life Technologies) to remove polyclonal and low-quality sequences. Further bioinformatic analysis was conducted using fastq data and custom workflow. Sequence reads < 180-bp were removed from the dataset. Leading and trailing low-quality bases were removed using Trimmomatic version 0.39 [33]. Fastx-Toolkit [34] was used to extract sequences with the minimum of 50% of bases with a quality score ≥ 25. Quality filtered sequences were separated into individual combinations of indexes in Geneious R11.1.5. Chimeras were removed using the default settings in UCHIME2 version 4.2.40 [35]. Next, the sequences were trimmed at 5′ and 3′ ends to exclude PCR primers.

Operational taxonomic unit (OTU) clustering was done in USEARCH version 11.0.667 [36]. Singletons (< 10 reads) were removed, and then OTUs were clustered from the sequences whose abundance exceeded a threshold of ten counts using the cluster_otus algorithm [37]. The OTU consensus sequences were compared to GenBank (www.ncbi.nlm.nih.gov) using BLASTN [38] optimized for highly similar sequences (megablast algorithm) [39]. The OTU consensus sequences were compared to GenBank using 97% identity threshold to determine tick species and 100% identities for identification of microsporidian species.

### Amplification and Sanger sequencing of the large fragment of 18S rDNA

A large fragment of the 18S rRNA gene, covering V1-V5 hypervariable regions, was amplified using V1F (CACCAGGTTGATTCTGCCTGAC) [40] and CM-V5R (TAANCAGCACAMTCCACTC) [29] primers. PCR reactions were prepared in two technical replicates, each in a total volume of 10 µl containing Hot FIREPol DNA Polymerase, 0.25 µM of each primer and 1 µl of template DNA. Amplification program was as follows: 12 min at 95 °C, followed by 35 cycles of 15 s at 95 °C, 1 min at 60 °C and 1 min at 72 °C, with a final extension step at 72 °C for 10 min. After amplification, technical replications were pooled, and 5 μl was analyzed by electrophoresis on 1.5% agarose gel stained with GelRed (Biotium, USA). Samples containing visible bands were purified with *Escherichia coli* exonuclease I and FastAP Alkaline Phosphatase (Thermo Scientific, USA) and sequenced using BigDye Terminator v3.1 Cycle Sequencing Kit and ABI Prism 3130XL Genetic Analyzer (Applied Biosystems, USA), following the manufacturer’s instructions. Sequence chromatograms were checked for accuracy in Geneious R11.1.5 (Biomatters Ltd.).

### Phylogenetic analysis

For phylogenetic analysis, we used all published 18S rRNA gene sequences assigned to the genus *Endoreticulatus*. According to Trzebny et al. [29], the genus *Cystosporogenes* was used as a close outgroup and a chytrid *Chytridiopsis typographi* (Chytridiopsida) was used to root the tree (Table 2). Sequences were aligned using the L–INS–i algorithm in MAFFT version 7.388 [58, 59] as implemented in Geneious R11.1.5. The final alignment consisted of 1436 nucleotide positions. The best fit model of DNA evolution (GTR + I + G) was chosen by PartitionFinder2 [60]. Phylogenetic trees were reconstructed using maximum likelihood in Garli version 2.0 [61] and Bayesian inference in MrBayes version 3.2.6 [62]. Each BI run of four independent chains was performed in 2 × 10,000,000 generations, and the trees were sampled every 1000 generations. The final consensus tree was generated after discarding the burn-in fraction of 25% of initial trees; the average standard deviation of split frequencies dropped below 0.003. Bootstrap support for the ML tree was calculated by using 10,000 data replicates as implemented in Garli. The tree was edited in FigTree version 1.4.4 [63] and further in Corel Draw X4.

**Table 2:** DNA sequences used in phylogenetic analysis

Microsporidium species	Host species	Host class	GenBank no	References
*Endoreticulatus bombycis*	*Bombyx mori*	Insecta	AY009115	[41]
*Endoreticulatus itiiti*	*Listronotus bonariensis*	Insecta	KJ755828	[42]
*Endoreticulatus poecilimonae*	*Poecilimon thoracicus*	Insecta	KJ755827	[43]
*Endoreticulatus schubergi*	*Lymantria dispar,* *Hyphantria cunea,* *Choristoneura fumiferana*	Insecta	L39109	[44, 45]
*Endoreticulatus* sp. CHW-2004 Bulgaria	*Lymantria dispar*	Insecta	AY502945	[46]
*Endoreticulatus* sp. CHW-2004 Taiwan	*Ocinara lida*	Insecta	AY502944	[46]
*Endoreticulatus* sp. CHW-2008 Austria	*Thaumetopoea processionea*	Insecta	EU260046	[47]
*Endoreticulatus* sp. isolate JMM 2007	*Loxostege sticticalis*	Insecta	MK929470	[48]
*Endoreticulatus* sp. Melnik	*Euproctis chrysorrhoea*	Insecta	KU900486	[43]
*Endoreticulatus* sp. PL01	*Ixodes ricinus*	Arachnida	MT911425	This study
*Endoreticulatus* sp. Shengzhou	*Bombyx mori*	Insecta	JN792450	[49]
*Endoreticulatus* sp. Sofia	*Euproctis chrysorrhoea*	Insecta	KU900485	[43]
*Endoreticulatus* sp. Zhenjiang	*Bombyx mori*	Insecta	FJ772431	[50]
*Endoreticulatus* sp. isolate EC	*Eilema complana*	Insecta	KY615713	[51]
Microsporidium sp. clone Chula Myositis 1	*Homo sapiens*	Mammalia	JN619406	[52]
*Cystosporogenes legeri*	*Lobesia botrana*	Insecta	AY233131	[53]
*Cystosporogenes operophterae*	*Operophtera brumata*	Insecta	AJ302320	[54]
*Cystosporogenes* sp. GKK-2009	*Agrilus anxius*	Insecta	GQ379703	[55]
*Cystosporogenes* sp. KCI-2	*Operophtera brumata*	Insecta	GU299511	[56]
*Chytridiopsis typographi*	*Ips typographus*	Branchiopoda	MH728789	[57]

### Statistical analysis

The chi-square test with Yates’ correction [64, 65] was used for statistical comparison between the microsporidian prevalence in feeding ticks and the ticks’ host as well as tick collection areas. The level of statistical significance was established at *P* < 0.05.

The statistical significances between microsporidian infection and developmental stage, year and collection area of unfed host-seeking ticks were analyzed using the chi-square test with Yates’ correction [64, 65], and differences were considered significant if *P* < 0.05.

## Results

Based on mini-COI sequence data, all analyzed ticks were unambiguously assigned to *I. ricinus*. The representative sequences are available in GenBank under accession nos. MZ970304–MZ970324.

Three feeding females (3.2%) collected from different dogs out for walks in the Rusalka area (Fig. 1; Additional file 2: Table S2) were positive for microsporidian 18S rDNA and shared the same DNA sequence (MT911430–MT911432), which was identical to sequences representing *E. intestinalis* found in humans (e.g. KM058742). However, the correlations between the microsporidian-positive ticks and both the tick host (*χ*^2^_Yest_ = 0.156, *df* = 1, *P* = 0.693) and tick collection areas (*χ*^2^_Yest_ = 0.443, *df* = 2, *P* = 0.801) were not statistically significant.

**Fig. 2:**
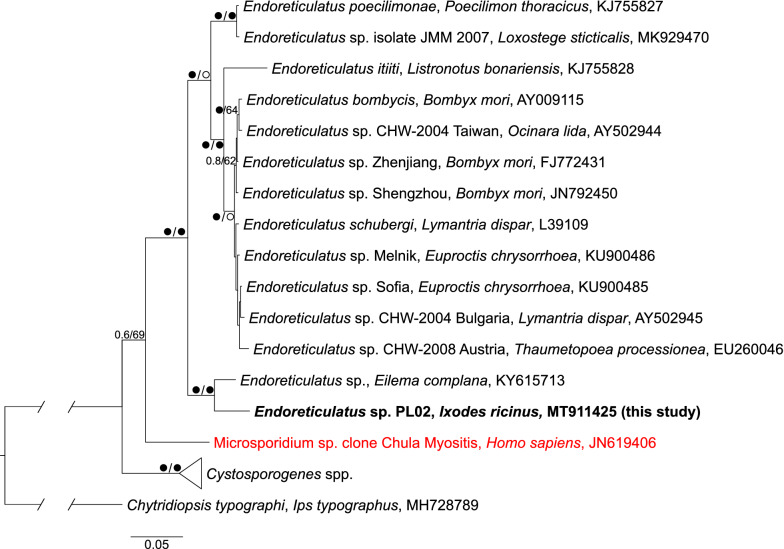
Phylogenetic relationships of *Endoreticulatus* 18S rDNA sequences inferred from Bayesian inference and maximum likelihood analyses. Values near branches show Bayesian posterior probabilities (PP) and bootstrap support values (BS) (PP/BS). Black circles: maximally supported; empty circles: supported > 0.95 PP and > 75% BS. The sequence found in this study is in bold. The uncultured human pathogenic microsporidium (JN619406.1) is in red

Microsporidian DNA was also detected in eight unfed host-seeking ticks (0.8%), including three males (1.1%), two females (0.7%) and three nymphs (0.7%) (Fig. 1; Additional file 1: Table S1). In this case also there was no correlation between tick developmental stage and microsporidian infections (*χ*^2^_Yest_ = 0.452, *df* = 3, *P*_Yest_ = 0.929). All microsporidian-positive samples shared the same 18S rDNA sequence (MT911422–MT911429) most similar to the group of sequences which originated from *Endoreticulatus* spp. (e.g. KY615713, LC467310, JN792450, AY009115). Phylogenetic analysis based on the longer fragment of the 18S rRNA gene (Fig. 2) revealed that the new sequence, named *Endoreticulatus* sp. PL02, is sister to the sequence derived from *Endoreticulatus* sp. found in *Eilema complana* (Lepidoptera) collected in Bulgaria [51].

None of the analyzed host-seeking ticks (*n* = 1070) were positive for any microsporidian species causing human microsporidiosis.

*Endoreticulatus* sp. PL02 was detected mainly in ticks collected near the Rusalka Lake: in three males and two females in 2017 and in two nymphs in 2018, while one infected nymph was identified in Morasko area in 2017. However, no correlation was found between *Endoreticulatus* sp. PL02 infection and the year of tick collection (*χ*^2^_Yest_ = 0.221, *df* = 1, *P*_Yest_ = 0.638) and the study area (*χ*^2^_Yest_ = 4.02, *df* = 5, *P*_Yest_ = 0.543).

## Discussion

Our data suggest that *I. ricinus* is not involved in transmission of zoonotic microsporidian species, because none of the analyzed host-seeking ticks was positive for any human pathogenic species. *Encephalitozoon intestinalis* was detected only in feeding ticks removed from dogs. There are numerous reports about the occurrence of human pathogenic microsporidians in dogs [66–69]. Similarly, *Encephalitozoon*-like microsporidians were found in *Amblyomma* and *Anocentor* ticks that had been collected from horses and then fed on rabbits [15, 16]. These parasites were observed in the midgut epithelial cells and acinus of the salivary gland in different phases of the host’s development, which suggests that this microsporidium may infect ticks. The authors suggested that the rabbit’s or horse’s blood meals were the source of infection [15, 16]. Presumably, in our study, the presence of *E. intestinalis* in engorged ticks resulted from their earlier feeding on dogs infected by microsporidians. Moreover, the lack of zoonotic microsporidians in unfed host-seeking nymphs and adults suggests that *E*. *intestinalis* is not transmitted transstadially.

Results of the phylogenetic analysis showed that *Endoreticulatus* sp. PL02 forms a clade with *Endoreticulatus* sp. recorded in an *E. complana* moth [51]. Both sequences were nested in the strongly supported clade grouping *Endoreticulatus* sequences found in insects (Fig. 2 and Table 2). In addition, our phylogenetic tree suggests that two sequences previously described as closely related to the *Endoreticulatus*, the Microsporidium sp. clones Chula Myositis 1 and Chula Keratitis 1, which caused microsporidian myositis or stromal keratitis in humans, probably represent a different genus, so far unknown from the complete 18S rRNA gene (Fig. 2) [70, 71].

Our data show that both the diversity and prevalence of microsporidians in *I. ricinus* are very low. Among unfed host-seeking ticks we noticed only one type of microsporidian 18S rDNA sequence. Considering that we detected *Endoreticulatus* sp. PL02 based on molecular analyses, the comparison with *N. slovaca* [20, 23], *Unikaryon* (*Nosema*) *ixodis* [17, 24] and *Nosema*-like [22] previously noticed in *I. ricinus* is unfeasible. Thus, further morphological and ultramicroscopy studies are needed to confirm that the *Endoreticulatus* sp. PL02 sequence found in this study represents a different species not yet recorded in ticks.

Low prevalence of microsporidians in *I*. *ricinus* found in this study corresponds well with the previous reports (Table 1). In the most extensive survey on the prevalence of microsporidians in ticks [18], none of about 6200 *I*. *ricinus* individuals were positive for these parasites, and only *N. slovaca* was found in 0.2% of about 3000 *D. reticulatus* ticks. Similar low prevalence was found in *D. reticulatus* (0.1%) and *I. ricinus* (0.5%) sampled in Hungary [20] and in *I. persulcatus* (1.6%) collected in the vicinity of St. Petersburg [21]. Additionally, the intensity of infection in *I. persulcatus* from St. Petersburg was very low and amounted to one spore per smear [21].

The presence of *Endoreticulatus* sp. PL02 in unfed host-seeking adults and nymphs suggests vertical transovarial transmission of this microsporidian species. Horizontal transmission is most frequently reported in Microsporidia [72–75]. Microsporidians transmitted vertically are typically less virulent than horizontally transmitted species and show higher specificity to the host. However, mortality of early developmental stages of hosts who acquired the parasite as a result of transovarial transmission is generally higher than for infections caused by species acquired during the host life span [60–62]. This mortality results from destruction of various host tissues and subsequent depletion of essential energy reserves necessary for pupation [72, 76–78]. Previous studies have shown that *Endoreticulatus* usually is transmitted horizontally and infects the midgut only; however, horizontal transmission also can occur in this genus [43, 76–78]. In *Lymantria dispar* (Lepidoptera), for example, it has been shown that *E. schubergi* was transmitted to offspring from females and males via the egg chorion [78]. Although our results suggest that *Endoreticulatus* sp. PL02 might spread vertically, the determination of the exact pathway of transovarial transmission of this species and its potential impact on *I*. *ricinus* nymph and larval mortality requires further detailed studies.

Ticks are exposed to microsporidians that infect the hosts they feed on. Consequently, these parasites also can infect the epithelial cells of the ticks’ midgut [15, 16]. However, in our study, the microsporidian species known to infect vertebrates were not observed in unfed host-seeking juvenile and adult ticks. Therefore, ticks must have evolved some mechanisms to overcome this infection. The low prevalence of microsporidians in ticks also suggests that ticks developed some unknown defense mechanisms against microsporidian infection. Some of them may involve the immune defense pathway to maintain the pathogens at the level, which does not impair their fitness and development [79–82]. The pro‐phenoloxidase (proPO) cascade has an important role in melanization reaction, which is the first immune response against pathogens [83, 84]. Cell-surface proteins of pathogens activate the conversion of proPO into active phenoloxidase (PO) [84]. Phenoloxidase activity is used to determine the host’s immune defense ability; higher PO activity levels mean higher resistance to pathogens [85–89]. Sokolova et al. [90] showed that during the infection of *Gryllus bimaculatus* by *Paranosema grylli*, the activity of PO expressed as the number of positively stained hemocytes was suppressed from 40–50% to 10–20%. Nevertheless, *N. grylli* does not suppress cellular reactions such as clamp formation and phagocytosis of liberated spores. In addition, Tokarev et al. [91] noticed that in *Locusta migratoria* and *G. bimaculatus* infected by *P. locustae* and *P. grylli*, respectively, microsporidians produce enlarged or malformed spores. These types of spore modifications were a result of abnormal sporogony and were noticed in the melanized sites, which indicates that melanization contributes to the abnormal development of microsporidians. Invertebrate immune systems are very heterogeneous [92]; therefore, assessment of how microsporidians influence the cellular immune response of ticks should be tested. These studies should also consider other factors, including tick gut microbiome composition and other pathogens that may co-occur with microsporidians.

## Conclusions

Our data suggest that *I. ricinus* is not involved in transmission of zoonotic microsporidian species. Moreover, we noticed a very low prevalence of these parasites in the tested ticks. Although we found *E. intestinalis* in engorged tick females, which indicates that ticks can acquire zoonotic microsporidians with their food, none of unfed tested ticks were positive for this microsporidian DNA. Moreover, a very low occurrence of the other microsporidian species in both fed and host-seeking ticks implies that mechanisms exist to defend ticks against infection with these parasites. The only microsporidian species found in unfed host-seeking ticks, *Endoreticulatus* sp. PL02, probably use *I*. *ricinus* as a specific host. Further studies are needed to determine the host specificity of *Endoreticulatus* sp. PL02 and potential defense mechanisms developed by ticks against microsporidian parasites.

## Supplementary Information

**Additional file 1: Table S1.** Characteristics of the questing ticks analyzed in this study (see Fig. 1 in the text).

**Additional file 2: Table S2.** Characteristics of feeding ticks analyzed in this study (see Fig. 1 in the text).

## Data Availability

Sequences generated in this study are available in GenBank under accession nos. MT911422–MT911432 and MZ970304–MZ970324. Additional details are available in Additional file 1: Table S1 and Additional file 2: Table S2. Sequence alignments and the remaining data that support the findings of this study are available from the corresponding author upon request.

## Abbreviations

18S rDNA: DNA coding small subunit ribosomal RNA gene
BS: Bootstrap support values
COI: Cytochrome *c* oxidase subunit I gene
CP: Cytadela park
ML: Area around Malta Lake
MO: Morasko
OTU: Operational taxonomic unit
PO: Phenoloxidase
PP: Bayesian posterior probabilities
proPO: Pro‐phenoloxidase
RL: Area around Rusalka Lake
SP: Solacki park
TP: Tysiaclecia park
