# Supplementary material for: Metabarcoding reveals low prevalence of microsporidian infections in castor bean tick (Ixodes ricinus)
Source: Parasit Vectors. 2022 Jan 15;15:26. doi: 10.1186/s13071-022-05150-9 (PMC8760655; doi:10.1186/s13071-022-05150-9)
Supplement: Supplementary file 1 — Additional file 1: Table S1. Characteristics of the questing ticks analyzed in this study (see Fig. 1 in the text). [file 13071_2022_5150_MOESM1_ESM.docx]

**Supplemental Information for:**

**Metabarcoding reveals low prevalence of microsporidian infections in castor bean tick (*Ixodes ricinus*)**

Artur Trzebny^1*^, Justyna Liberska^1^, Anna Slodkowicz-Kowalska^2^, Miroslawa Dabert^1^

^1^ Molecular Biology Techniques Laboratory, Faculty of Biology, Adam Mickiewicz University, Poznan, Poland

^2^ Department of Biology and Medical Parasitology, Faculty of Medicine I, University of Medical Sciences, Poznan, Poland

*** Corresponding author:
Artur Trzebny**: Molecular Biology Techniques Laboratory, Faculty of Biology, Adam Mickiewicz University, Poznan, Poland; e-mail: arturtrzebny@amu.edu.pl

**Table S1.** Characteristics of the questing ticks analyzed in this study (see Figure 1 in the body text).

| **Characteristics of the collection places** | | | **Characteristics of the ticks**  **(infected/tested)**  **[year of collection a microsporidian-positive ticks]** | | | | |
| --- | --- | --- | --- | --- | --- | --- | --- |
| **Collection place** | **Description** | **Coordinates** | **Female** | **Male** | **Larvae** | **Nymph** | **Total** |
| Area around the Malta Lake  (ML) | Area around the Malta Lake with a range of 67.5 ha. This area is inhabited by numerous animals such as foxes, hedgehogs, moles, mice, rats, shrew and weasels. Area around the Malta Lake includes recreation centers, rope parks, golf courses, etc. Numerous concerts, festivals and sports events take place every year.  All ticks from this area were collected in 2017. | N 52.401944 E 16.970556 | 0/65 | 0/63 | 0/16 | 0/109 | 0/253 |
| Morasko  (MO) | Poorly urbanized of >1000 ha area located on the northern border of the city. This park is inhabited by numerous animals such as, foxes, hedgehogs, mice, polecats, roe deer, weasels and wild boars. Numerous hiking, walking, cycling and horseback riding trails run through the this area.  All ticks from this area were collected in 2017. | N 52.468425 E 16.925980 | 0/15 | 0/9 | 0/0 | **1/17**  **[2017]** | **1/41** |
| Cytadela park  (CP) | The largest park in Poznan, with an area of 100 ha. This park is inhabited by numerous animals such as bats, foxes, hedgehogs, moles, mice, polecats, rats, shrew, squirrels and weasels. This park contains museum, cemeteries, and the remains of fortifications. Every year, exhibitions, concerts and family festivities are held here.  All ticks from this park were collected in 2017. | N 52.421111 E 16.935278 | 0/24 | 0/41 | 0/0 | 0/4 | 0/69 |
| Area around the Rusalka Lake  (RL) | Area around the Rusalka lake with a range of 36.7 ha. This area is inhabited by numerous animals such as foxes, hedgehogs, mice, roe deer shrew, squirrels and wild boars. The route around the lake is about 3.5 km surrounded by forests with many walking paths and artificial beaches. Every year, a numerous sports and recreational events take place here.  In this area 98 female, 89 male and 29 nymph were collected in 2017. The remaining ticks were collected in 2018. | N 52.426389 E 16.877778 | **2/170**  **[2017]** | **3/153**  **[2017]** | 0/1 | **2/313**  **[2018]** | **7/637** |
| Solacki park  (SP) | A 14.6 ha park with a large meadow and playgrounds surrounded by rows of trees and bushes. This area is inhabited by numerous animals such as foxes, hedgehogs, mice, rats and squirrels. In addition, numerous walking and cycling paths run through this place.  All ticks from this park were collected in 2017. | N 52.422597 E 16.901897 | 0/15 | 0/14 | 0/13 | 0/17 | 0/59 |
| Tysiaclecia park  (TP) | A 26.3 ha park directly next to Malta Lake. This park is inhabited by numerous animals such as foxes, hedgehogs, moles, mice, rats, shrew and weasels. The area includes an amphitheater with band shell. Therefore, every year numerous concerts and festivals are held.  All ticks from this park were collected in 2017. | N 52.406944 E 16.967778 | 0/2 | 0/6 | 0/0 | 0/3 | 0/11 |
| **Total** | | | **2/291** | **3/286** | **0/30** | **3/463** | **8/1070** |
