# Supplementary material for: Metabarcoding reveals low prevalence of microsporidian infections in castor bean tick (Ixodes ricinus)
Source: Parasit Vectors. 2022 Jan 15;15:26. doi: 10.1186/s13071-022-05150-9 (PMC8760655; doi:10.1186/s13071-022-05150-9)
Supplement: Supplementary file 2 — Additional file 2: Table S2. Characteristics of feeding ticks analyzed in this study (see Fig. 1 in the text). [file 13071_2022_5150_MOESM2_ESM.docx]

**Supplemental Information for:**

**Metabarcoding reveals low prevalence of microsporidian infections in castor bean tick (*Ixodes ricinus*)**

Artur Trzebny^1*^, Justyna Liberska^1^, Anna Slodkowicz-Kowalska^2^, Miroslawa Dabert^1^

^1^ Molecular Biology Techniques Laboratory, Faculty of Biology, Adam Mickiewicz University, Poznan, Poland

^2^ Department of Biology and Medical Parasitology, Faculty of Medicine I, University of Medical Sciences, Poznan, Poland

*** Corresponding author:
Artur Trzebny**: Molecular Biology Techniques Laboratory, Faculty of Biology, Adam Mickiewicz University, Poznan, Poland; e-mail: arturtrzebny@amu.edu.pl

**Table S2.** Characteristics of feeding ticks analyzed in this study (see Figure in the body text).

| **Collection place** | **Description** | **Coordinates** | **Females**  **(infected/tested)** | **Total** |
| --- | --- | --- | --- | --- |
| Area around the Rusalka Lake  (RL) | Ticks collected from 31 dogs out for walks in the area around Rusalka Lake.  All ticks were collected in 2013. | N 52.426389 E 16.877778 | **3/60** | **3/60** |
| Area around the Malta Lake  (ML) | Specialist Veterinary Clinic. Ticks collected from two cats out for walks around Malta Lake. Additionally, one cat out for walks in Ostrow Wielkopolski (N 51.649418, E 17.815689), located about 120 km (0.62 miles) from the area.  All ticks were collected in 2016. | N 52.400406 E 16.962844 | 0/34 | **0/34** |
| **Total** | | | **3/94** | **3/94** |
